# A Description of the Temporal Pattern of Out-of-Pocket Expenditure Related to Iranian Healthcare Services during 1995–2014

**Published:** 2018-10

**Authors:** Masoud BEHZADIFAR, Tina BEYRANVAND, Mehdi JAFARI, Meysam BEHZADIFAR, Masood TAHERI MIRGHAED, Mariano MARTINI, Nicola Luigi BRAGAZZI

**Affiliations:** 1. Health Management and Economics Research Center, Iran University of Medical Sciences, Tehran, Iran; 2. Dept. of Health Services Management, School of Health Management and Information Sciences, Iran University of Medical Sciences, Tehran, Iran; 3. Social Determinants of Health Research Center, Lorestan University of Medical Sciences, Khorramabad, Iran; 4. Dept. of Health Sciences (DISSAL), Section of History of Medicine and Ethics, University of Genoa, Genoa, Italy; 5. Dept. of Health Sciences (DISSAL), School of Public Health, University of Genoa, Genoa, Italy

**Keywords:** Healthcare reforms, Health economics, Iran, Out-of-pocket expenditure

## Abstract

**Background:**

Out-of-pocket (OOP) expenditure directly impacts on poverty and household welfare, especially when there is a decline in healthcare financing. This study was aimed to describe the temporal pattern of OOP expenditures related to Iranian healthcare services during 1995–2014.

**Methods:**

For describing the trend of OOP spending in Iran, the database of the World Bank was mined for the period under study. Further, the trend analysis has been complemented by an exhaustive and comprehensive review of the extant literature.

**Results:**

From 1995 to 2014, out-of-pocket decreased from 53.59% to 47.8% of the total health expenditure, probably because of the different health reforms implemented throughout the years. However, out-of-pocket expenditure in Iran remains higher than the world average (roughly 3 times higher)

**Conclusion:**

It is an onus of the Iranian government to make serious attempts in order to reduce out-of-pocket expenditure, as well as to protect particularly poor and vulnerable subjects against catastrophic health expenditure. In order to ensure an equitable and affordable access to the healthcare system, decision- and policy-makers in Iran should implement a review of health care costs, insurance tariffs, and healthcare services packages covered by insurance organizations as well as introduce a progressive tax-based financing scheme as soon as possible.

## Introduction

Iran is a country characterized by an impressive extension of more than 160000 km^2^, and, as such, is one of the most populous countries in the Middle East (1). Decades of wars have resulted in considerable losses, with the death of 204795 Iranian lives and with severely damaged economic infrastructure (1). Further, Iran has faced with a freezing in bilateral relationships with the USA, followed by the USA sanctions on Iran for its alleged support of international terrorism and the embargo policy, with a consequent decline of foreign investment in the country. For these reasons, the Iranian government has implemented different socio-economic and health reforms, with Iran being actually classified by the World Bank among the countries with low-medium income. Nowadays, Iran has completed both the demographic and epidemiological transitions and the pattern of the burden of disease has definitely shifted towards noncommunicable diseases, with a strong focus of the Iranian healthcare system on prevention (1).

In 2000, Iran ranks 58^th^ in healthcare and 93^rd^ in health-system performance (2). Recently, in 2016, Bloomberg News has ranked Iran 30^th^ as one of the most efficient healthcare systems, ahead of the United States and Brazil (3). Whilst the primary care is financed by the Iranian government, different insurance schemes finance secondary/tertiary care services (1). According to the official estimates, Iran spends approximately 5.7%–6% of its gross domestic product (GDP), corresponding to US$ 432, per capita on healthcare delivery, much more than the expenses of other Middle Eastern and North African countries (1). Expenditure for healthcare has increased from 1.66% of GDP in 1971 to 5.5% of GDP in 2000 (1).

However, despite its achievements and its strong points including excellent health outcomes, Iranian healthcare system is plagued by some weakness, including out-of-pocket (OOP) expenditure, addressed in the following paragraphs.

### Access to health-care as a constitutional right

From a jurisdictional standpoint, access to healthcare in Iran is a constitutional right, universally guaranteed, as stated by the Articles 29 and 43 (4). On the other hand, the application of this right from the Iranian government has to take into account that economic resources are limited. As such, economic constraints and the principle of a “responsible budget” limit public expenditure, profoundly impacting on accessibility and affordability of healthcare services.

Ensuring a fair delivery of healthcare services undoubtedly constitutes one of the most important priorities for Iran, as a dynamic, young country (with more than 50% of the entire population aged <20 yr), based on a health and social welfare, both in terms of societal development and sustainability (1). To achieve this ambitious goal, the government, which every year allocates enormous resources to the health sector, is trying to increase, as well, the dissemination of health education programs, aimed at promoting a healthy lifestyle, health literacy, and health awareness, in the effort to attain high health indicators and standards. Generally speaking, based on the type of health care service required and benefit, there are different levels of financial pressure imposed on the social strata (5).

### Ensuring financial protection in healthcare systems

Protecting people against the financial risks potentially deriving from the achievement of important goals of health policy and health sector is the main onus of a government. The aim was also influenced by the precise way according to which the health system is financed (6).

The lack of an adequate protection against the possible dangers and risks arising from the relevant financial cost of health care could push millions of people towards poverty. As a consequence, they could not afford anymore any healthcare service, and in the impossibility of curing, they should accept the suffering generated by their disease, not seeking medical assistance even though in need and, as such, reporting a low level of quality. This issue is of urgent and crucial importance not only in poor countries but as well in rich developed contexts and realities (7).

The concept of financial protection in health, increasing attention has attracted a huge body of scholarly research as well as attention of decision- and policy-makers. In particular, international organizations and governments are focusing on the high costs generated by healthcare systems and making efforts to mitigate the risks, supporting particularly poor and vulnerable individuals (8).

### Out-of-pocket expenditure

OOP expenditure represents one of the simplest, yet most effective ways to pay less for the delivery of healthcare services. Individuals pay directly the provider for the services. OOP expenditure directly impacts on poverty and household welfare, especially when there is a decline in healthcare financing (9). In many developing countries, OOP is particularly widespread and this represents a serious obstacle to an equitable access to health care services (10). Practices including balance billing and informal or under the table payment explain the relevant OOP spending (1).

We aimed to evaluate the temporal pattern of OOP expenditures related to Iranian healthcare services during 1995–2014.

## Methods

For describing the time series of OOP expenditures related to Iranian healthcare services during 1995–2014, data were collected from the database of the World Bank (11). In particular, OOP expenditure has been defined and computed as any direct outlay by households to healthcare practitioners and deliverers, and, as such, is a part of private health expenditure.

Further, the trend analysis has been complemented by an exhaustive and comprehensive review of the extant literature.

## Results

**Fig. 1:**
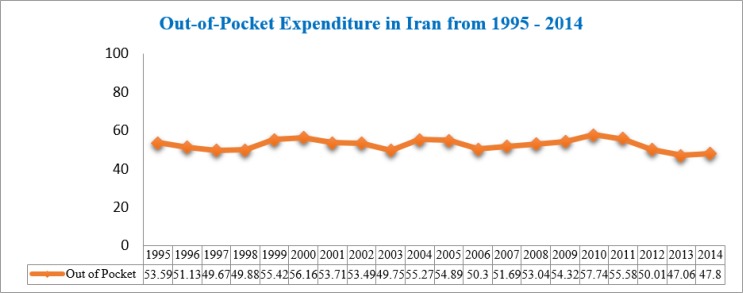
Out-of-pocket health expenditure (computed as percentage of total expenditure on health) in Iran in the period 1995–2014

In 2014, according to the World Bank, the average OOP expenditure was 18.15% of the total health expenditure. From 1980 to 2001 in Iran, OOP spending has considerably increased, with an estimated 2% of households facing catastrophic health care spending (that is to say, allocation of more than 40% of the households’ income to healthcare services). From 1995 to 2014, it decreased, instead, from 53.59% to 47.8% (Fig. 1), probably because of the different health reforms implemented throughout the years.

## Discussion

On the basis of the data obtained from the World Bank database, OOP expenditure remains a serious concern in Iran and it is an onus of the Iranian government to make serious attempts in order to reduce it. OOP expenditure in Iran, indeed, remains higher than the world average (being roughly 3 times higher).

Rising health-related costs and OOP expenditure in healthcare systems are, indeed, associated with dissatisfaction and social injustice and inequality. Further, OOP expenditure is associated with poor health outcomes, such as high infant mortality rate (12).

Exploring the determinants of OOP expenditure, out-of-pocket expenditure did not statistically correlate with patient age but with gender. In particular, males tended to spend more than females. Diagnostic services induced high volumes of OOP expenses (13). Spending on health care services had considerably increased from 2002 to 2008, with OOP expenditure rate stable or slightly higher. In particular, in the last year of the study period, health-related expenditure was 6.40% and 6.35% of the total expenses for urban and rural areas, respectively (14).

In 2008, a cross-sectional multi-stage survey computed that household spending for health care services was 201,496,172 million Rials (+34.4% with respect to 2007). The percentage of out-of-pocket expenditure was 53.79% of the total health expenditure (15).

Considering the period from 1998 to 2012, a descriptive, cross-sectional study was performed. The average inequality index was 0.48 for both rural and urban regions, whilst the mean of OOP expenditure was 0.51 and 0.50 in rural and urban regions, respectively. The mean values of Kakwani index (OKI) were −0.005 and −0.018 for rural and urban areas, respectively (16).

The effects of Iran’s fourth development program (2006–2011) on Iranian household healthcare payments have been descriptively analyzed. The overall OKI was progressive (0.013) and regressive (0.012) in urban and rural areas, with a negative and positive impact on income redistribution, respectively (17).

In 2012, a cross-sectional study showed that the equity in health finance index and the income redistributive effect index were 0.84 and 0.48, respectively, for medically insured households, whilst for households, without medical insurance, they were 0.83 and 0.25. The incidence of catastrophic health expenditure was 2.4% and 4.0% for urban and rural households, respectively, with the change in the figures of extremely impoverished households at 0.4% and 2.0%. The percentage of catastrophic health expenditure was computed to be 2.8% in medically insured households, and 3.0% in households without, with a difference in the figure of households characterized by extreme impoverishment (below the poverty line) of 0.008 and 0.011, respectively (18).

The fifth 5-year economic, social and cultural plan was analyzed (2011–2016). In particular, the plan aimed at reducing the OOP spending related to Iranian healthcare services by 30% (19).

In 2013, a cross-sectional study based in Tehran found that the mean OOP expenses yielded a figure of US$ 44.33 and of US$ 1,861.11, for outpatients and inpatients, respectively. The authors computed also the concentration index for OOP expenses, which showed a level of inequity (20).

In another investigation, the same group performed a cross-sectional study recruiting 772 families of patients. Approximately 21% of households were found to experience catastrophic health expenditure, while the incidence of impoverishment was 2.8%. Having members under 6 yr or over 60 yr in household, household size, employment of household head, households’ income quintile, presence of disabled members and the educational level of the household’s head predicted the impact of out-of-pocket expenditure (21).

After the establishment of Mr. Rouhani’s government in Iran (settled on 3^rd^ Aug 2013 and incumbent until 3^rd^ Aug 2017), more effective healthcare reform plan has been advocated, aiming at ensuring an adequate and appropriate level of financial protection of particularly vulnerable subjects to high and rising health-related costs, while preserving and improving quality of care and increasing access to healthcare services (22).

Concerning the period from 2013 to 2014, a cross-sectional study recruiting 19,437 rural and 18,888 urban households was performed. The percentage of subjects living below the poverty line was in the range 0.50%–14.3% and 0.48–13.27% for households in the rural and urban areas, respectively, with a percentage of catastrophic health expenses varying from 9.62% to 18.72% and from 8.80% to 17.74% (23).

The impact assessment of the Health Sector Evolution Plan (HSEP) implemented since 2014, based on a survey of 663 households carried out in 2015, has found that the rate of households facing catastrophic health expenditure was 4.8%. Comparing this values with previous figures, the authors concluded that the implementation of the HSEP had contributed to reduce catastrophic health expenditure rate at the household level (24).

Summarizing, Iranian health policy- and decision-makers have succeeded in increasing access to health care services, better integrating healthcare services, improving quality of care in remote and rural areas (25), and reducing, at least to some extent, OOP expenditure, represents a major concern in low- and middle-income countries, as documented by a recently published systematic review (26).

## Conclusion

In Iran, different socio-economic and health plans have been approved and launched throughout the years. However, despite some successes and achievements, still many steps have to be taken to achieve the desired goal.

OOP expenditure still represents a major concern, as documented by our description of the temporal pattern of OOP expenditure related to Iranian healthcare services during 1995–2014.

In order to ensure equity and sustainability of the Iranian healthcare system, decision- and policy-makers should implement an effective, robust review of health care costs, insurance tariffs, and healthcare services packages covered by insurance organizations as soon as possible. Further, they should consider progressive and not regressive measures and initiatives, such as a progressive tax-based financing scheme.

## Ethical considerations

Ethical issues (Including plagiarism, informed consent, misconduct, data fabrication and/or falsification, double publication and/or submission, redundancy, etc.) have been completely observed by the authors.
